# Ionic Liquid-Based Grapeseed Oil Emulsion for Enhanced Anti-Wrinkle Treatment

**DOI:** 10.3390/ph17101273

**Published:** 2024-09-26

**Authors:** Bo Yang, Xu Zhang, Liguo Zhang, Jinjin Wu, Wei Wang, Qiaomei Huang, Zhenyuan Wang, Jichuan Zhang, Tongjie Xu, Chengyu Wu, Jiaheng Zhang

**Affiliations:** 1 Sauvage Laboratory for Smart Materials, Harbin Institute of Technology, Shenzhen 518055, China; 15196008769@163.com (B.Y.); zhangjichuan@hit.edu.cn (J.Z.); 2 Harbin Fuerjia Technology Co., Ltd., Harbin 150000, China; 18846780205@163.com (X.Z.); zlg007@188.com (L.Z.); 13946133882@139.com (W.W.); 18903654065@139.com (T.X.); 3 Shenzhen Shinehigh Innovation Technology, Co., Ltd., Shenzhen 518056, China; wujinjin@shinehigh.cn (J.W.); huangqiaomei@shinehigh.cn (Q.H.); wangzhenyuan@shinehigh.cn (Z.W.); 4 College of Pharmacy, Shenzhen Technology University, Shenzhen 518118, China

**Keywords:** ionic liquid, grape seed oil, transdermal penetration, enhanced efficacy, antiwrinkle treatment

## Abstract

Objectives: To address the poor efficacy and percutaneous penetration of grape seed oil, ionic liquids and nanotechnology were combined to prepare a grape seed oil emulsion. Methods: A novel Menthol-CoQ10 ionic liquid and ionic liquid based grapeseed oil emulsion were prepared and confirmed. Results: The average size of the grapeseed oil emulsion was 218 nm, and its zeta potential was −33.5 mV. The ionic liquid-based grape seed oil emulsion exhibited a transdermal penetration effect 4.63-fold higher than that of ordinary grape seed oil emulsion. Ionic liquid also displayed enhanced efficiency both in vitro and in vivo. It significantly inhibited the production of DPPH free radicals and tyrosinase, inhibited melanin and matrix metalloproteinase-1 (MMP-1) produced by cells, and promoted type I collagen expression in fibroblasts. After 28 days of continuous use, the grapeseed oil emulsion improved the water content of the stratum corneum and the rate of transepidermal water loss, enhanced the firmness and elasticity of the skin, and significantly improved the total number and length of under-eye lines, tail lines, nasolabial folds, and marionette lines on the face. Conclusions: Menthol-CoQ10 ionic liquid is a promising functional excipient for both transdermal delivery increase and efficient enhancement. Ionic liquid and nanotechnology for grape seed oil facial mask displayed significantly enhanced efficacy and permeability.

## 1. Introduction

Linoleic acid is an essential fatty acid involved in the synthesis of cellular phospholipids and is an important component of cell and mitochondrial membranes. Grape seed oil is rich in linoleic acid [1], tocopherols and their derivatives, phytosterols, and phenols [2], which can resist ultraviolet radiation, effectively remove oxidative free radicals, and inhibit ultraviolet damage to the skin. It can effectively inhibit the oxidation of oil esters and has a protective effect on skin collagen and elastin [3]. Additionally, grapeseed oil has a whitening effect that can eliminate pigmentation caused by skin aging, promote wound healing, and improve skin metabolism [4]. Grapeseed essential oil has a soothing effect on nerves, stabilizes mood, and can provide a better soothing effect on skin [5].

Natural ionic liquids are widely used in pharmaceutical research because of their good biodegradability; biocompatibility; sustainability; excellent extraction efficiency; and ability to promote drug solubility [6], permeability [7,8], stability, and absorption. Choline chloride is an ionic liquid formed from citric acid, proline, organic acids, or sugars that increases the solubility of cinnarizine (CIN), tolbutamide (TBM), nimesulide (NIM), and domperidone (DOM) in water [9]. NADES, formed by choline chloride, glycerol and citric acid, has better bioavailability than organic solvents for anthocyanin glycosides extracted from crude blueberry extracts, which can increase the absorption efficiency of anthocyanin glycosides [10]. Flavonoids (rutin) in Sophora japonica flower buds were extracted with a water content of 20% choline chloride/triethylene glycol solution, and the extraction rate was 194.17 ± 2.31 mg/g [11]. NADES, which are composed of lactic acid, glucose, and water, have the highest performance for phenolic extraction and greatly improve the stability of phenol, hydroxytyrosol (Hty), uteolin (Lut), and total anthocyanins [12]. NADES formed with menthol and fatty acids can respond to microbial invasion and biofilm detachment without altering normal keratinocyte proliferation and migration, as validated in wound healing and epidermal repair, helping to reduce cellular stress and inflammation by controlling ROS production [13]. Due to the abundance of nutrients, oil esters lose nutrients or experience oxidative rancidity during their shelf life, and choosing the appropriate ionic liquid can enhance the stability of oil esters and prolong their shelf life.

Transdermal administration involves the application of a drug to the epidermis of the skin, where the drug reaches the target or site of the injury by absorption by the skin. The transdermal absorption efficiency of active substances in the skincare process is the key point of transdermal penetration. Breakthroughs have been made in the research of transdermal technology such as nano, hydration [14], microneedle [15], and conductivity. Nanoparticles are nanosized particles that can be designed into different structures using wall materials, such as solid–lipid nanoparticles (SLNs), nanostructured lipid carriers (NLCs), polymer nanocarriers (e.g., dendritic polymers), inorganic nanoparticles, and hybrid carriers [16]. They are designed and encapsulated to deliver drugs to the dermal layer of the skin. Nanomaterials can improve drug stability, prolong its release time in the skin, enhance drug penetration, and deliver the target drug to the target location [17]. Chitosan-coated nanocurcumin formulations have a larger particle size, a higher sedimentation-volume ratio, a higher solubility and dissolution rate, and colloidal storage stability, which can significantly increase the curcumin content that penetrates the skin [18].

The stratum corneum plays an important role in protecting the skin from damage, preventing excessive moisture loss, and preventing the penetration of active ingredients [19]. The moisture in the mask can hydrate together with the stratum corneum, fully moisturize the skin, soften the keratin, and form a high concentration difference between the inside and outside through penetration and hydration; the active ingredients in the mask can effectively penetrate the skin and promote cell metabolism. With the addition of science and technology, thin-film liquids and thin-film materials have been applied to various scientific and technological technologies, such as nanotechnology, electrospinning technology [20], freeze-drying technology [21], and electronic technology [22], to develop different types of mask products, and the development of all technologies is also closely related to improving the transdermal penetration and absorption of active ingredients.

The combination of ionic liquids, nanotechnology, and facial masks for grapeseed oil can enhance the efficacy and improve the stability during storage. The use of nanotechnology to load compound components and deliver them through the skin can effectively enhance the transdermal absorption efficiency and bioavailability of the active substance, and the penetration mode of different dosage forms can also have different effects on penetration. 

Therefore, we aimed to achieve grapeseed oil emulsion with high permeability and increased efficiency in vitro and in vivo. The efficient bioavailability and efficacy of grapeseed oil was achieved by combining Menthol-CoQ10 ionic liquid and nanotechnology, supplemented by facial masks. The antioxidant activity, inhibition of tyrosinase, cytotoxicity, and cellular production of melanin, collagen, and MMP-1 were thoroughly investigated, and the clinical efficacy of the components was verified.

## 2. Results and Discussion

### 2.1. Characterization of the Ionic Liquid

**Figure 1:**
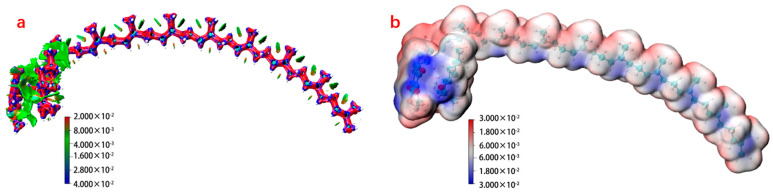
Molecular simulation of bonding in Menthol-CoQ10 ionic liquids, weak interaction (**a**) and electrostatic potential (**b**).

The ionic liquid of the Menthol-CoQ10 was a eutectic structure formed by the intermolecular forces between L-menthol and Coenzyme Q10. Molecular dynamics simulations combined with the multifunction analysis program Multiwfn showed (Figure 1a) that there was a large intermolecular force between menthol and Coenzyme Q10, which was formed by dispersion dominated by weak hydrogen bonding forces. The hydroxyl group in l-menthol and the benzoquinone group in Coenzyme Q10 formed weak hydrogen bonds, exhibiting low electrostatic potential energy at this spatial location (Figure 1b).

**Figure 2:**
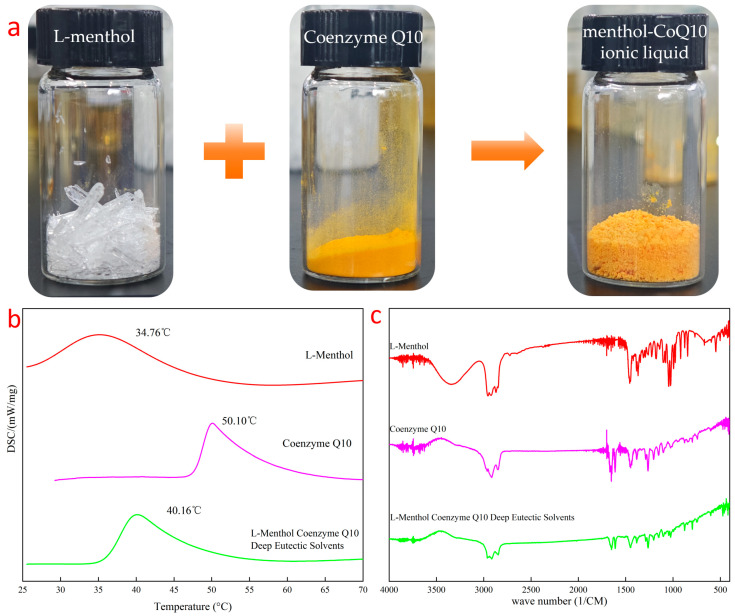
Image of Methol-CoQ10 ionic liquid (**a**), DSC curve (**b**), and FTIR spectrum (**c**) of the ionic liquid Menthol-CoQ10.

Menthol-CoQ10 ionic liquid had a yellow, fluffy powder appearance (Figure 2a). Changes in microscopic spatial interactions can affect the way substances are stacked in crystals and have an impact on enthalpy and entropy during the melting process, which in turn can lead to changes in the physical properties of substances [23]. The ionic liquid of the menthol Coenzyme Q10 exhibited a maximum endothermic temperature of 40.16 °C on Differential Scanning Calorimeter (DSC), which is between the maximum endothermic peaks of the two precursors and has a different endothermic temperature from the two precursors (Figure 2b). The intermolecular interaction of the ionic liquids of Menthol-CoQ10 was investigated by FTIR spectroscopy (Figure 2c). L-menthol exhibited a hydroxyl peak structure of cyclohexanol at 3346 cm^−1^ and 2800–3000 cm^−1^. However, after formation of the cocrystal, combined with the electrostatic potential ESP diagram (Figure 1b), the hydroxyl peak was masked by the strong *p*-π conjugated electron cloud of benzoquinone, manifested in the infrared spectrum without its characteristic peak hydroxyl. L-menthol was firmly encapsulated by Coenzyme Q10, which showed the disappearance of not only the characteristic hydroxyl peaks in the infrared spectrum but also the methyl and methylene peaks. Therefore, the cocrystals showed an infrared spectral peak structure similar to that of Coenzyme Q10 in the infrared spectrum. NMR analysis (Figure 1) showed that the hydrogen in the hydroxyl group in L-menthol was completely shielded without showing peak information, and the phenyl methyl of Coenzyme Q10 showed a blue shift (0.48 Hz), demonstrating that there was an interaction between L-menthol and Coenzyme Q10, resulting in some spatial changes between the two. These results indicate the successful preparation of the ionic liquid of the menthol Coenzyme Q10.

### 2.2. Characterization and Stability of GSO-ILE

**Figure 3:**
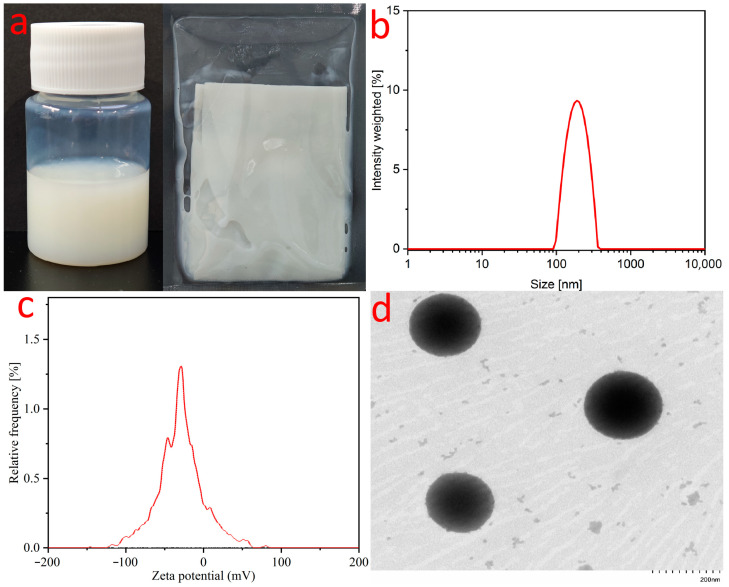
GSO-ILE appearance (**a**), particle size (**b**), zeta potential (**c**), and TEM view (**d**).

Stability studies characterize the steady-state change behavior of emulsions over a certain period of time, and the behavior of substances without stratification precipitation, oxidation discoloration, and reduced content can be demonstrated to have good stability and shelf life [24]. GSO-ILE was formed by mixing Menthol-CoQ10 ionic liquid with grapeseed oil to dissolve it into an ionic liquid phase and homogeneously emulsify it with a high-pressure emulsion matrix (Figure 3a). GSO-ILE had average particle size of 193.59 nm, a PDI of 0.182, and an average potential of −33.5 mV (Figure 3b,c). The Transmission Electron Microscope (TEM) showed that GSO-ILE had a small particle size with spherical shape.

The results of the GSO-ILE stability analysis showed good centrifugal stability in the centrifugal experiment at 2000 rpm for 30 min, indicating that the particles of the system were well suspended. The results of the destructive test of 3 months showed that GSO-ILE was stable under different time and temperature conditions. There was no aggregation, precipitation, oxidative discoloration, demulsification, or stratification. The results showed no significant change in the particle size and zeta potential of GSO-ILE in these accelerated experiments (Figures S3 and S4 in the Supplementary Materials), indicating that GSO-ILE was relatively stable in structure. The test results for Coenzyme Q10 in GSO-ILE during the same period (Figure S5 in the Supplementary Materials) found that the content of Coenzyme Q10 in different time conditions was above 90%, indicating good stability. The physical properties and contents of the prepared grapeseed oil emulsions were relatively stable [25].

### 2.3. In Vitro Transdermal Release of GSO-ILE

The transdermal permeability model characterizes the strength of the absorption of active substances in the 3D cell skin and the transdermal situation based on the content and distribution of active substances. Fluorescent-labeled actives are typically characterized [26]. The 3D cell skin model and the Franz flow cell method are the main methods for the osmotic diffusion testing of commonly used active substances to evaluate the penetration of GSO-ILE. The results of the transdermal fluorescence test of GSO-ILE with coumarin-6 at 2 and 4 h showed that GSO-ILE was distributed into the deep layers and widely distributed in the true epidermis, whereas the grapeseed oil emulsion only remained on the upper side of the 3D cell skin model. With increasing time, the permeability of GSO-ILE increased significantly, indicating that the osmotic absorption effect of GSO-ILE was significantly stronger than that of ordinary grapeseed oil emulsions.

**Figure 4:**
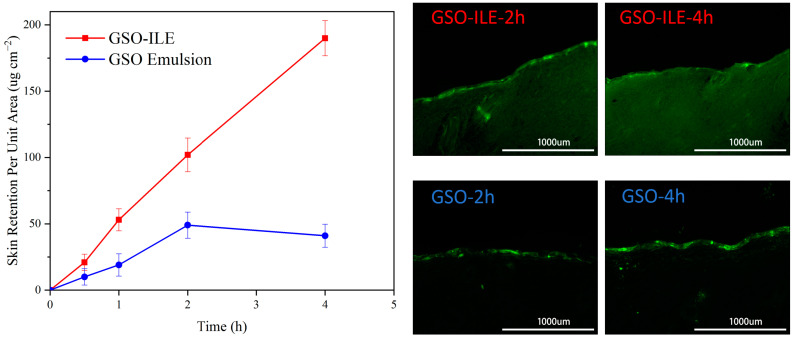
Penetration of Coenzyme Q10 in the 3D cell skin model of GSO-ILE and emulsion of grapeseed oil (**left**) and fluorescence distribution (**right**).

The fluorescence distribution intensity can be used for the qualitative analysis of the transdermal permeation efficiency, whereas the permeation of active substances can be quantified by high-performance liquid chromatography, which has a positive effect on improving the transdermal permeation of GSO-ILE through a combination of qualitative and quantitative analyses. The results of high-performance liquid chromatography (HPLC) measurement of Coenzyme Q10 at different time points showed that the microemulsion of grapeseed oil was stably released within 4 h, the release rate was basically the same (Figure 4), and the content of Coenzyme Q10 in the 3D cell skin model was proportional to time. However, the release rate of the grapeseed oil emulsion slowed after 2 h and decreased at 4 h, indicating that the transdermal efficiency was low due to the difficulty of transdermal transdermalization, and the skin had no absorption, or the absorption rate was lower than the release rate after 2 h, resulting in a decrease in the intradermal Coenzyme Q10 content. For comparison, the absorption rate multipliers were found to be 2.8, 2.1, and 4.6 times at 1, 2, and 4 h, respectively. The results indicated that the GSO-ILE could continuously penetrate and be release into the skin for a long time, and the saturation of the GSO-ILE in the skin did not reach the limit. In this process, Menthol-CoQ10 ionic liquid could be a major reason for enhanced permeation.

### 2.4. DPPH Free Radical Scavenging Effect

**Figure 5:**
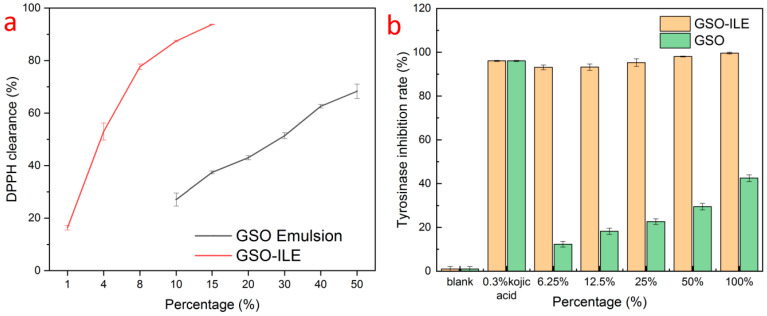
(**a**) DPPH free radical scavenging rate and (**b**) tyrosinase inhibition rate of GSO-ILE.

The DPPH method is widely used [27] to detect the antioxidant capacity of the preparation. The results of DPPH free radical scavenging by GSO-ILE showed that the DPPH scavenging rate was 93.7 ± 0.1% at 15% volume concentration (Figure 5a), while the DPPH scavenging rate was 16.4 ± 0.9% at only 1% concentration. In contrast, grapeseed oil exhibited only 27.1 ± 2.5–68.3 ± 2.7% DPPH radical scavenging at concentrations of 10–50%. The above comparison shows that GSO-ILE has an excellent free radical scavenging effect on DPPH, which is significantly better than that of grapeseed oil, and has a significant antioxidant effect. In GSO-ILE, the component of CoQ10 in the ionic liquid could also make a certain contribution in this DPPH test.

### 2.5. Tyrosinase Inhibition

Tyrosinase plays an important role in skincare activities [28], as an oxidative restriction enzyme that regulates melanin production, and as a key control node in the synthesis reaction of melanin synthesis [29]. In the tyrosinase inhibition study, GSO-ILE with different percentages showed a good tyrosinase inhibition rate, and the inhibition rate of 6.2% GSO-ILE on tyrosinase conversion of dopa to dopaquinone also reached 93.1 ± 1.1% (Figure 5b). However, the inhibitory effect of a single grapeseed oil on tyrosinase conversion to dopa was only 42.5 ± 1.5% at a 100% concentration, and only 12.3 ± 1.3% at a 6.2% concentration. Grapeseed oil inhibited dopaquinone synthesis by tyrosinase; however, its effect was much weaker than that of GSO-ILE.

### 2.6. Effect on the Viability of Various Types of Cells

**Figure 6:**
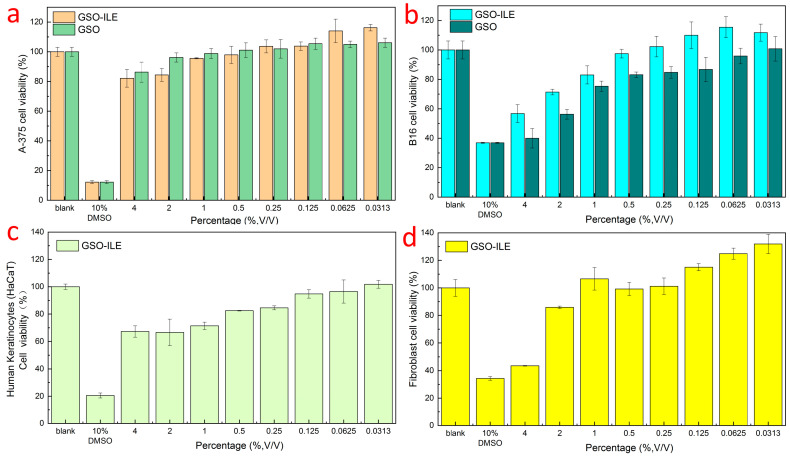
Survival rate of GSO-ILE on various of cell types, human melanoma cells (**a**), mouse melanoma cells (**b**), keratinocytes (**c**), and fibroblasts (**d**).

Cell viability refers to the survival of cells after drug application, and the ratio of viable cells is used to evaluate the safety and efficacy of a drug or composition and to aid in development and evaluation to determine the accuracy and efficacy of subsequent efficacy evaluations [30]. Both GSO-ILE and grapeseed oil exhibited high safety profiles against human melanoma cells (A-375), with cell viability above 90% at a concentration of 1% (Figure 6a). GSO-ILE showed a cell survival rate of 0.5% for mouse melanoma cells (B16) at a concentration of more than 90%, whereas the corresponding concentration of grapeseed oil with cell viability of more than 90% was only 0.062% (Figure 6b). At the same concentration, the grapeseed oil microemulsion had a greater effect on cell survival than the grapeseed oil alone, indicating that the grapeseed oil microemulsion had better cytocompatibility and lower cytotoxicity. GSO-ILE had good cell survival for keratinocytes and fibroblasts (Figure 6c,d) and good cell compatibility at high concentrations (0.25–2%). Subsequent cell efficacy verification tests could be carried out at high concentrations.

### 2.7. Melanoma Cells (B16) Test for Melanin Content

**Figure 7:**
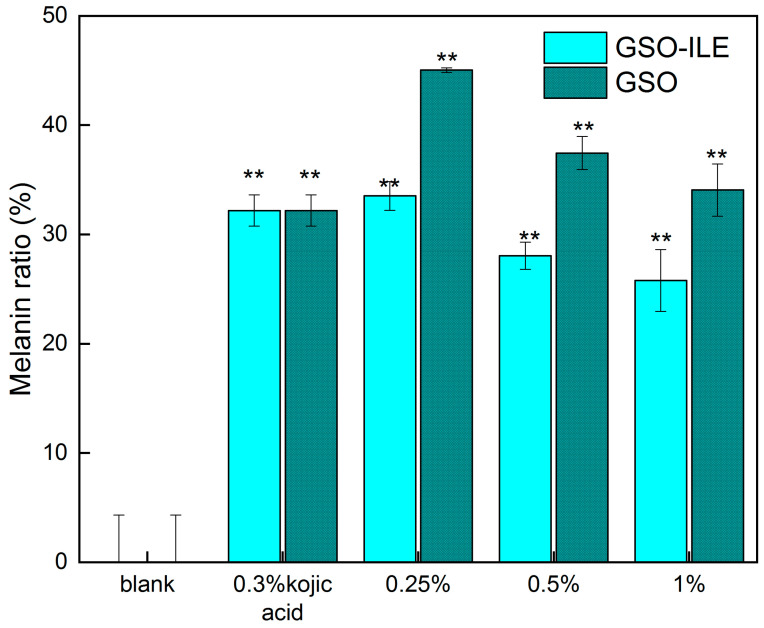
Inhibition of melanin production by GSO-ILE in melanoma cells (B16). Statistical analysis was performed using the *t*-test. Compared to the blank group, *p*-value < 0.01 is expressed as “**”.

Melanin, as an amino acid-derived protist biological pigment, is usually produced by pigment mother cells in order to protect the skin against ultraviolet rays, which can convert 99% of harmful ultraviolet rays into harmless heat, protect the survival of organisms, and delay the aging of organisms, especially the skin; thus, melanin is also widely distributed in all parts of the skin. In addition to ultraviolet light, which can affect melanin production, chemical drugs are one of the factors used to measure the amount of melanin in melanoma cells to evaluate the efficacy of the sample whitening. GSO-ILE and grapeseed oil exhibited good cell survival in the concentration range of 0.25–0.50%, and the test in this concentration range not only ensured efficacy but also ensured the effectiveness of efficacy (Figure 6b). Evidently, different concentrations of GSO-ILE and grapeseed oil could significantly change the melanin content in melanoma cells. The inhibition rates of melanin production by melanoma cells at concentrations of GSO-ILE of 0.25% and 0.50% concentrations of GSO-ILE were 66.5 ± 1.3% and 71.9 ± 1.2%, respectively (Figure 7). The inhibition rates of melanin production by melanoma cells were 62.6 ± 1.5% and 65.9 ± 2.4%, respectively. This indicates that grapeseed oil has a better inhibitory effect on melanin synthesis in melanoma cells, confirming conclusions from previous reports [31], and it also has an inhibitory effect on melanin synthesis in melanoma cells which is better than that of grapeseed oil.

### 2.8. Testing of Type I Collagen and Matrix Metalloproteinase-1 Content in Fibroblasts

**Figure 8:**
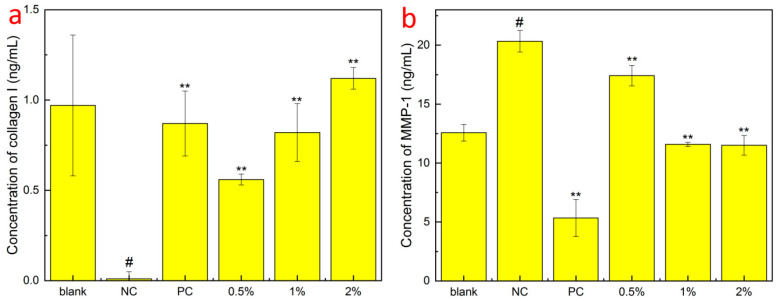
Collagen type I production (**a**) and inhibition of MMP-1 (**b**) by GSO-ILE in fibroblasts. Statistical analysis was performed using the *t*-test. NC was denoted by “#” compared to the blank group, with a *p*-value of <0.05 as “#”. The significance of the PC and sample groups was denoted by “**”—*p*-value of <0.01, compared to the blank group.

Collagen is generally considered to be the supporting structure of the dermis; its network structure provides protection and elasticity for the skin, and between the fibers, there is a large amount of water, extracellular matrix, and functional cells, which is an important biochemical reaction site of the skin, providing water and nutrients to the epidermal layer, and human dermal fibroblasts maintain skin elasticity by synthesizing human type I collagen. It can be seen that compared to the NC group, GSO-ILE had different degrees of significant promotion of type I collagen synthesis by fibroblasts in the concentration range of 0.5–2.0% (Figure 8a). The 0.5% and 2.0% grapeseed oil microemulsions increased human type I collagen by 0.56 ± 0.03 ng/mL and 1.12 ± 0.06 ng/mL, respectively, which were 64.4% and 128.7% of the positive control. The content of human type I collagen increased with increasing concentration, indicating that GSO-ILE promotes the synthesis of human type I collagen and has a good cell-tightening effect.

Skin aging is macrocosmic, manifested by the loss of skin elasticity, wrinkles, and dullness, while microscopic manifestations include changes in the structure of the epidermis and dermis and the loss of functional components such as proteins and hyaluronic acid. Stimulation by external factors can cause fibroblasts to secrete more inflammatory factors, which can increase the production of matrix metalloproteinases (MMP) and improve the collagen breakdown activity of MMP-1, leading to a gradual loss of type I collagen in human skin [32]. MMP-1 is the most important enzyme for the degradation of type I and type III collagen. When MMP-1 is overexpressed, the components of the extracellular matrix are specifically degraded, the normal structure of collagen and elastic fibers is destroyed, and the active substances are lost. The secretion of type I collagen and matrix metalloproteinase-1 by GSO-ILE into fibroblasts at different concentrations was tested at concentrations based on a fibroblast viability of ≥90% (Figure 6d). The results (Figure 8b) showed that compared with the NC group, the concentration of 0.5–2.0% GSO-ILE had different degrees of inhibition of MMP-1 synthesis by fibroblasts. The content of MMP-1 synthesis in fibroblasts inhibited by 1% GSO-ILE was 11.584 ± 0.175 mg/mL, which was 43.03% lower than that of the negative control. These results indicate that different concentrations of GSO-ILE have a good anti-wrinkle effect on the cells.

### 2.9. Evaluation of Clinical Trials

Clinical efficacy verification is the process of characterizing the efficacy of a drug or formula in the process of use and making an objective evaluation conclusion on the efficacy claim of a product under laboratory conditions in accordance with prescribed methods and procedures through subjective evaluation, objective measurement, and statistical analysis of human experimental results. 

The cosmetic patch test serves as the stage of safety verification of human clinical experiments to ensure the safety and reliability of drugs or formulas in the human body and to avoid allergens and irritants that cause great harm, similar to the skin test before the use of antibiotics [33,34]. The results showed that 32 test subjects had negative patch reactions at different times after applying the grapeseed oil emulsion mask, with redness caused by partial non-response or single patch allergy, but no red in the center. The blank matrix plus membrane cloth group also showed negative results, which indicated that GSO-ILE, matrix, and membrane cloth were not irritating, indicating that the grapeseed oil emulsion mask has high safety and applicability and would not cause irritation or allergic reactions to the user. Based on this conclusion, human clinical efficacy verification tests should be conducted.

**Figure 9:**
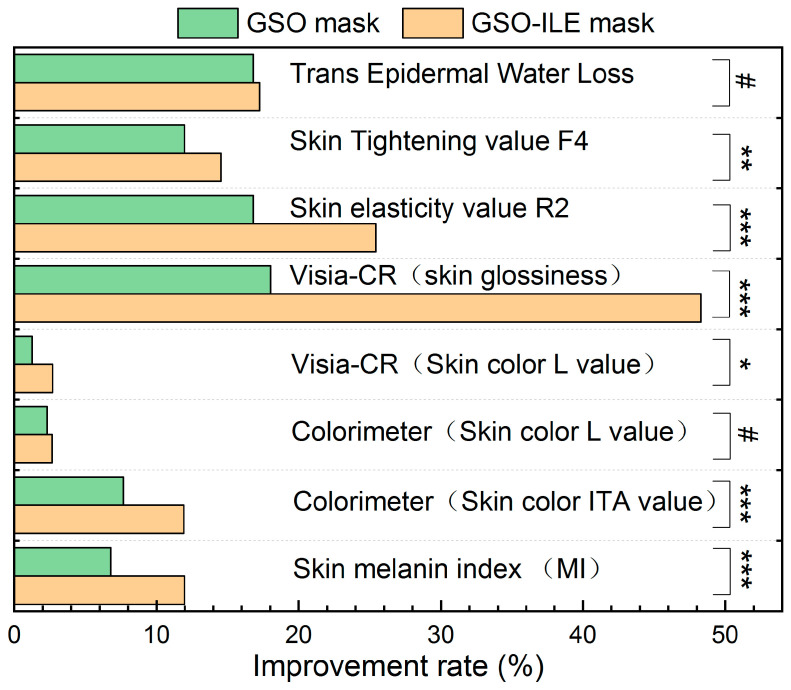
Clinical trials of GSO-ILE masks and grapeseed oil masks improved outcomes after 28 days of use. All statistics were expressed as mean ± SD (*n* = 30). Statistical analysis was performed using the *t*-test. “#” means no statistical difference, *p* value is greater than or equal to 0.05, *p* value less than 0.05 means significant difference (“*” means 0.01 ≤ *p* < 0.05, “**” means 0.001 ≤ *p* < 0.01, “***” means *p* < 0.001).

In the 28-day test of the GSO-ILE mask and GSO mask, the transepidermal water loss of the skin was significantly improved compared with the initial (day 0), and the improvement rate of GSO-ILE mask reached 17.25% (Figure 9), but there was no significant difference between the GSO-ILE mask and the GSO mask. Similarly, the GSO-ILE mask and GSO mask had significant improvement effects on skin elasticity value R2 and skin firmness value F4, and the GSO-ILE mask could increase elasticity value by 25.43% and firmness by 14.53%, which was significantly better than the GSO mask. In the whitening effect of the GSO-ILE mask, after 28 days of use, the skin melanin index improved by 11.97%, the colorimeter (skin tone ITA value) improved by 11.92%, the Visia-CR (skin tone L value) improvement rate was 2.69, and the skin radiance improvement using Visia-CR test skin radiance reached 48.28%, which was significantly better than the whitening effect of the grapeseed oil emulsion mask. The above studies have shown that the GSO-ILE mask can significantly improve the firmness, elasticity and repair effect of the skin and has a stronger whitening effect.

**Figure 10:**
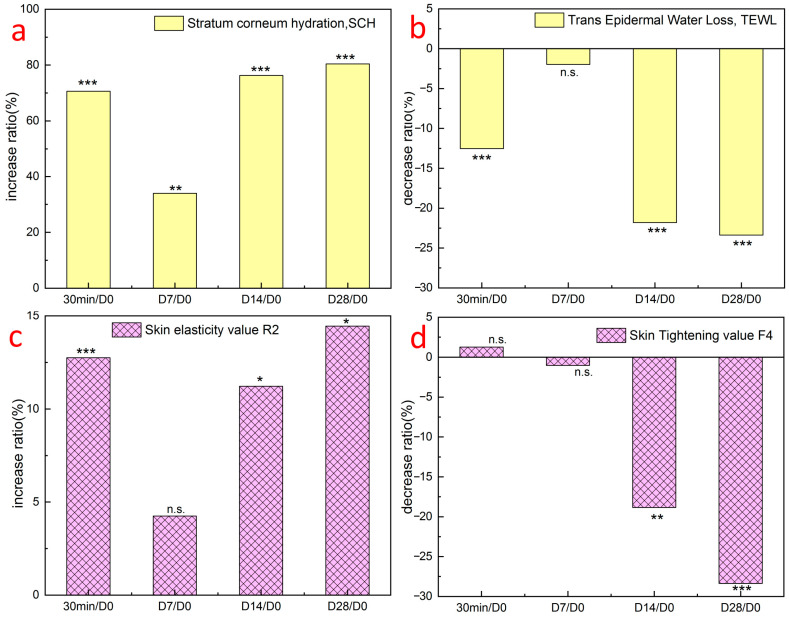
Results of clinical trials using the GSO-ILE mask at different times: cuticle water content (**a**), percutaneous water loss (**b**), elasticity R2 (**c**), and compactness F4 (**d**). All statistical data are represented as mean ± SD (*n* = 30). Statistical analysis was performed using the *t*-test. “n.s.” means no statistical difference, *p* value is greater than or equal to 0.05, *p* value less than 0.05 means significant difference (“*” means 0.01 ≤ *p* < 0.05, “**” means 0.001 ≤ *p* < 0.01, “***” means *p* < 0.001).

The results of the clinical efficacy verification of the GSO-ILE mask showed that the moisture content of the stratum corneum (Figure 10a) and skin elasticity value R2 (Figure 10c) increased by 70.7% and 12.8%, respectively, within 30 min and by 80.4% and 14.5%, respectively, after 28 days of long-term treatment. The transepidermal water loss rate (Figure 10b) and skin firmness F4 (Figure 10d) decreased by 12.5% and 18.8% at 30 min, respectively, and by 23.4% and 28.4% on day 28, respectively, indicating that the GSO-ILE mask significantly enhanced the moisture content of the stratum corneum, improved transepidermal water loss, and improved skin elasticity and firmness.

**Figure 11:**
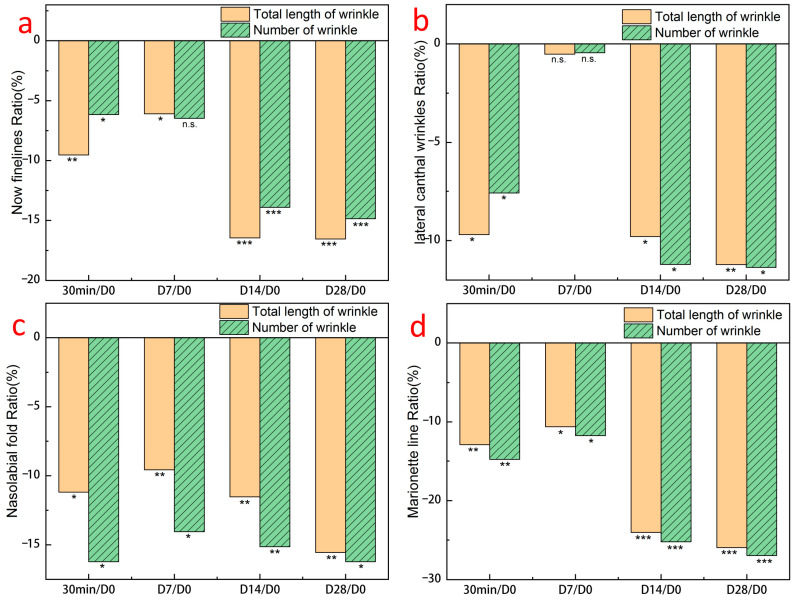
Clinical trial results of facial wrinkles at different times: eye (**a**), tail (**b**), decree (**c**), puppet (**d**). All statistical data are represented as mean ± SD (*n* = 30). Statistical analysis was performed using the *t*-test. “n.s.” means no statistical difference, *p* value is greater than or equal to 0.05, *p* value less than 0.05 means significant difference (“*” means 0.01 ≤ *p* < 0.05, “**” means 0.001 ≤ *p* < 0.01, “***” means *p* < 0.001).

**Figure 12:**
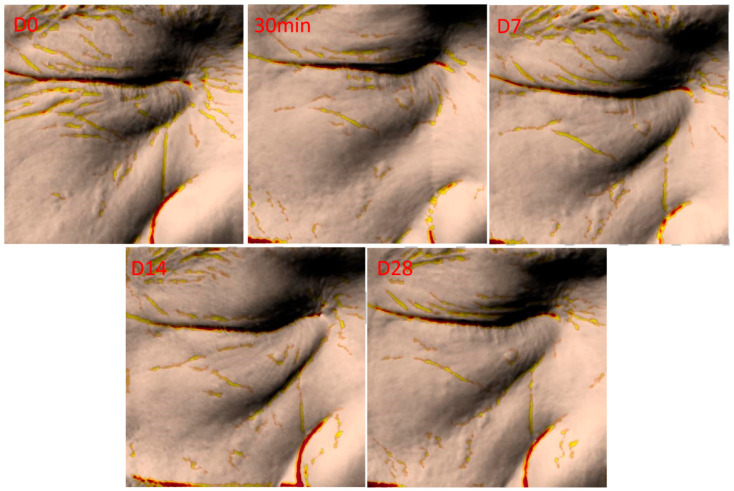
Representative images of improved facial skin wrinkles after 28 days of using a grapeseed oil microemulsion mask.

After using the GSO-ILE mask, the total length of wrinkles under the eyes, eye-tail lines, nasolabial folds, and marionette lines (Figure 11a–d) within 30 min decreased by 9.5, 9.7, 11.2, and 9.7%, respectively, and the total number decreased by 6.2, 7.6, 16.2, and 14.8%, respectively, indicating that the GSO-ILE mask had a significant short-term anti-wrinkle effect on the length and number of wrinkles in various parts of the face. After using the grapeseed oil microemulsion mask for 28 days, the total length of various wrinkles decreased by 16.5, 11.2, 15.6, and 26.0%, and the total number of various wrinkles decreased by 14.9, 11.4, 16.2, and 27.0%, respectively, indicating that the GSO-ILE mask has a significant long-term anti-wrinkle effect on the length and number of wrinkles in various parts of the face. Figure 12 shows the changes in facial wrinkles after a volunteer used the GSO-ILE mask for 28 days. It can be seen that the yellow mark is used on the furrow part of the picture, and the number of yellow furrow stripes decreases and narrows with the increase of the number of uses, indicating that the number and volume of wrinkles can be significantly reduced with the use of GSO-ILE mask for a long time. Overall, clinical verification of human efficacy proved that the GSO-ILE mask has good moisturizing, firming, repairing, and antiwrinkle effects. Although there was no significant improvement in eye wrinkles in the short term (<7 days), the GSO-ILE mask could significantly improve the stratum corneum hydration, transepidermal water loss, skin elasticity, and firmness, and it had a positive effect on under-eye wrinkles, eye tail lines, nasolabial folds, and marionette lines.

## 3. Materials and Methods

### 3.1. Materials

Grapeseed oil (GSO), L-menthol, Coenzyme Q10, methanol, ethanol, DPPH, horseradish peroxidase (HRP), coumarin-6, tyrosinase, levodopa, pyroic acid, and PBS solutions were purchased from Shanghai Aladdin Biochemical Technology Co., Ltd. (Shanghai, Shanghai, China). The emulsion matrix was provided by Shenzhen Shinehigh Innovation Technology Co., Ltd. (Shenzhen, Guangdong, China); 3D EpiKutis skin (Keratinocytes isolated from skin tissue of Chinese were used as seed cells), keratinocytes, fibroblasts, and melanoma cells were obtained from Guangdong Biocell biotechnology Co., Ltd. (Guangzhou, Guangdong, China).

### 3.2. Preparation of OIL-ILNPS

At 50 °C, equimolar menthol and Coenzyme Q10 were mixed in a round-bottom flask and stirred in the dark for 2 h to obtain Menthol-Coenzyme Q10 (Menthol-CoQ10) ionic liquid. Then, 0.5 g of Menthol-CoQ10 and 80 g of grapeseed oil were evenly dispersed to obtain ionic liquid grapeseed oil (GSO-IL). A further 9 g of GSO-IL was mixed with 91 g of emulsion matrix, and at 50 °C, a 600 bar high-pressure homogenization (ATS Technology, Shanghai, China) was used to obtain the grapeseed oil ionic liquid emulsion (GSO-ILE), which was then combined with the membrane cloth to form a grapeseed oil ionic liquid emulsion mask.

### 3.3. Investigation of the Ionic Liquid

To determine whether there was an interaction force between L-menthol and Coenzyme Q10, we simulated the electrostatic potential (ESP) and weak interaction force (IRI) between L-menthol and Coenzyme Q10 using the wave function analysis program Multiwfn (Beijing Kein Research Center for Natural Sciences, Beijing, China). Specifically, this was performed by constructing the menthol and Coenzyme Q10 configurations, selecting the appropriate force field, minimizing the energy of the system through software simulation, equalizing the process, and performing kinetic simulation.

The thermal properties of Menthol-CoQ10 ionic liquids were analyzed using a differential scanning calorimeter DSC 250 (Shimadzu, Tokyo, Japan). The Menthol-CoQ10 ionic liquid (approximately 5 mg) was placed in a standard flat bottom aluminum pan and heated from −50 to 100 °C in a nitrogen atmosphere at a rate of 10 °C/min.

To characterize hydrogen bonding in Menthol-CoQ10 ionic liquids, Fourier transform infrared (FTIR) spectroscopy IRSpirit (Shimadzu, Tokyo, Japan) was used to evaluate functional group interactions, and nuclear magnetic resonance hydrogen spectroscopy JNM-ECZ600R/S1 (JEOL, Tokyo, Japan) was used to study hydrogen atom coupling in Menthol-CoQ10 ionic liquids.

### 3.4. Characterization of Grapeseed Oil Emulsion

#### 3.4.1. Measurements of Particle Size, Polymer Dispersity Index (PDI), and Zeta Potential

GSO-ILE was dispersed in deionized water, and particle size, PDI, and zeta potential were determined using a polydisperse particle size analyzer (ZetaLitesizer 500, Anton Paar GmbH, Graz, Austria). Each measurement was repeated three times.

#### 3.4.2. High-Performance Liquid Chromatography (HPLC)

To determine the stability of GSO-ILE, HPLC (Agilent1260, California, CA, USA) was used to determine the Coenzyme Q10 content. Each measurement was repeated three times. HPLC conditions were Inertsil HILIC LC Columns 250 mm × 4.6 mm, 5 μm, 275 nm, 1 mL/min, absolute ethanol–methanol (65:35). The method validated a linear relationship (R^2^ = 0.997), good reproducibility (RSD 0.02%), and determined the limit of detection (LOQ of 0.04 μg/mL). All tests were performed in triplicate replicates, and the mean and standard deviation were calculated.

### 3.5. In Vitro Transdermal Release Test

GSO-ILE and traditional emulsions were prepared with equivalent concentrations of grapeseed oil, and in vitro permeation tests were performed using 3D cell skin model (Boxi Biotechnology Co., Ltd., Guangzhou, China). GSO-ILE was used as the experimental group, grapeseed oil emulsion was used as the control group.

In vitro transdermal testing was performed in accordance with FDA requirements [35]. The Franz diffusion flow cell was used to simulate the in vivo penetration test at 320 rpm and 32 °C, and the receiving cell was used with 0.9% NaCl aqueous solution. The Q10 Coenzyme content in the 3D cell skin extract at specific time points was detected by HPLC. Each measurement was repeated three times; the experiment was carried out three times in parallel.

### 3.6. DPPH Free Radical Scavenging Test

The sample tubes (T), sample background (T0), DPPH tubes (C), and solvent background (C0) were prepared with three parallel tubes per sample. Next, 1 mL of the same concentration of the sample solution was added to the sample tube (T) and sample background (T0), and 2 mL of the sample solvent was added to the sample background (T0) and solvent background (C0) to mix well. Next, 1 mL was added to sample tubes (T) and DPPH tubes (C), were shaken gently, and allowed to stand for 5 min at 25 °C. Each tube of the reaction solution was placed in a 1 cm cuvette, and the absorbance value was determined at 517 nm for three parallel experiments.

(1) $$DPPH\;clearance\;rate\;\%\;=(1-\frac{\left(T-T_{0}\right)}{\left(C-C_{0}\right)})\times 100\%$$

### 3.7. Tyrosinase Inhibition

Tyrosinase inhibition experiment-biochemical method was used to prepare the samples to be measured, the control group (a) was added with 10 μL PBS buffer and 90 μL tyrosinase, the control background group (b) was supplemented with 100 μL PBS buffer, the sample group (c) was supplemented with 10 μL of the test solution and 90 μL of tyrosinase, the sample background group (d) was added with 10 μL of the test solution and 90 μL of PBS buffer, and the positive control group was added with 10 μL of Kojic acid and 90 μL of tyrosinase. The five groups were incubated at 37 °C for 30 min, 100 μL of L-dopa was added, and the absorbance value was measured at 475 nm wavelength, and the experiment was carried out three times in parallel.

(2) $$Inhibition\;rate\%\;=\frac{\left(A_{a}-A_{b})-(A_{c}-A_{d}\right)}{\left(A_{a}-A_{b}\right)}\times 100\%$$

### 3.8. Viability of Various Types of Cells

Different cell lines (purchased from Boxi) were seeded in 96-well plates according to the seeding density listed in Table 1, in a volume of 100 μL per well, and incubated until adherent in an incubator (37 °C, 5% CO_2_). Treatments were added when the cell plating rate reached 40–60%. The control group was supplemented with 200 μL of culture medium containing 10% PBS per well; the positive control was supplemented with 200 μL of culture medium containing 10% DMSO per well. The sample group was supplemented with 200 μL of culture medium containing different concentrations of GSO-ILE per well, no cell seeding was added to the zero group, and only 200 μL of cell culture medium was added. After the dosing was completed, the 96-well plate was placed in an incubator (37 °C, 5% CO_2_) and cultured with three replicate wells set at each concentration.

After 24 h of cell incubation, the supernatant was discarded, MTT working solution (0.5 mg/mL) was added and incubated at 37 °C for 2 h, the supernatant was discarded at the end of incubation, 100 μL DMSO was added to each well, and the OD value was read at 490 nm

(3) $$RGR\%=\frac{Test\;{OD}_{450nm}}{Neg\;{OD}_{450nm}}\times 100\%$$

### 3.9. Melanoma Cells (B16) Test for Melanin 

Logarithmic growth phase cells were collected at a cell density of 2 × 10^5^/well seeded in 24-well plates and after 24 h of culture in an incubator (37 °C, 5% CO_2_). To determine cytotoxicity, 0.3% kojic acid was added to the positive control group (a), different concentrations of GSO-ILE were added to the experimental group (b), the untreated cells were used as a blank control (c), and three parallels were established in each group. After 24 h of incubation in an incubator (37 °C, 5% CO_2_) after dosing, the supernatant was discarded, 1 mL of 1 M NaOH containing 10% DMSO was added, incubated in an oven at a constant temperature in an oven for 1 h, and 200 μL per well was transferred to a 96-well plate after returning to 25 °C. Then, 1 M NaOH with 10% DMSO was used as blank control, the absorbance value was read at 405 nm, and the relative inhibition of cytomelanin was calculated:

(4) $$Inhibition\;rate\%=1-\frac{\left(A_{b}-A_{c}\right)}{\left(A_{a}-A_{c}\right)}\times 100\%$$

### 3.10. Fibroblast Type I Collagen and Matrix Metalloproteinase-1 Content Test

Human skin fibroblasts were seeded in 24-well plates at a density of 8 × 10^4^ cells/well and incubated overnight in an incubator (37 °C, 5% CO_2_). When the cell plating rate in the 24-well plate reached 40–60%, 1 mL of cell culture medium was added to each group of blank control and negative control; 1mL of culture medium containing 100 μg/mL of vitamin C and 7 μg/mL of vitamin E was added to each positive control well, and 1 mL of GSO-ILE containing different concentrations was added to each well of the experimental group. Twenty-four hours after administration, the negative and positive controls received a total dose of 9 J/cm^2^ of UVA radiation, and the blank control was placed in the same environment but with a UVA radiation dose of 0 J/cm^2^. After 24 h of incubation, the supernatant was collected and stored cryostat in an EP tube at −80 °C.

The type I collagen content was detected and analyzed according to the instructions for human type I collagen (ColI) ELISA kit. Specifically, the required slats were removed from the aluminum film bag after 20 min of 25 °C equilibration, standard wells and test holes were set, and 50 μL of standard products of different concentrations were added to the standard wells; 10 μL of the test sample was added to the test well, and then 40 μL of sample diluent was added. In addition to the blank wells, 100 μL of horseradish peroxidase (HRP) labeled detection antibody was added to each well of the standard well and the test well, the reaction well was sealed with a sealing film, incubated at a constant temperature of 37 °C for 1 h; the plate was washed 5 times, 50 μL of substrate A and B were added to each well, 50 μL of stop solution was added after incubation at 37 °C for 15 min in the dark, and the absorbance value data at 450 nm was read immediately.

Type I collagen content was detected and analyzed according to the instructions of the MMP-1 mouse matrix metalloproteinase-1 assay ELISA kit. Specifically, 100 μL of detection sample was added; 100 μL of detection solution A was added after incubation at 37 °C for 1 h and 100 μL of detection solution A was added and the plate was washed 3 times after incubation at 37 °C for 1 h, 100 μL of detection solution B was added, and 100 μL of 37 °C was incubated for 0.5 h. The plate was washed five times before 90 μL of TMB substrate and 50 μL of stop solution were added, absorbance value data at 450 nm were read immediately, and three repeat wells were set at each concentration.

### 3.11. Evaluation of Clinical Trial Safety

To evaluate the actual effect of the GSO-ILE mask, a clinical trial was conducted to explore its moisturizing, firming, and antiwrinkle skin effects. A patch test for sealing human skin was performed in accordance with the requirements of the “Safety and Technical Standards for Cosmetics” (2015 Edition). Patches were closed with a GSO-ILE emulsion mask for 24 h and skin reactions were observed after 0.5, 24, and 48 h to determine the scoring level.

### 3.12. Evaluation of the Efficacy of Clinical Trials

Based on the passing of the clinical safety evaluation test, determined using clinical medical theory, to evaluate the actual efficacy of the GSO-ILE mask and GSO mask on human skin, a non-invasive skin testing instrument was used to evaluate changes in skin related parameters of subjects after cosmetics use.

According to clinical medical theory, 32 healthy volunteers aged 30–65 years were selected. They were not sensitive to commonly used cosmetics, had not participated in other clinical research projects in the past three months, and had passed the clinical evaluation criteria. They were not sensitive to commonly used cosmetics; did not participate in other products or behaviors with similar effects or affect the test results during the test; did not use cosmetics, perfumes, fragrances, etc. at the time of detection; pregnant and lactating women participated in the test, filled in the informed consent form, and pass the lactate test during the test, cleaned the face, and waited in a constant temperature and humidity room until the test began. Subjects used the test product daily and had skin parameters tested before the test, at minute 30, day 7, day 14, and day 28. This clinical study was conducted in accordance with the ethical principles of the Declaration of Helsinki of the World Medical Association and the International Code of Ethics for Biomedical Research with Human Subjects. The ethical internal lot numbers are 202212JC04 and 202302JC03, and the subject informed consent numbers are HZP-SH-SOP-COM016-F01 and Q/WP-SHACDDGX-WIO-001-R01 A/0. When test samples were used, it was forbidden to use products with similar efficacy, and the conditions at the test site were not changed.

The specific method was as follows: after cleansing, unfold the mask so that the mask overlaps the facial skin, remove the mask after 10–15 min, and gently massage the face to absorb the remaining essence.

The moisture content of the stratum corneum of the facial skin was measured using a skin moisture tester (CM825, Courage + Khazaka Electronic GmbH, Cologne, Germany), and the transepidermal water loss rate of the facial skin was measured using a transepithelial water loss tester (VAPOSCAN AS-VT100RS, ASCH Japan Co., Ltd., Tokyo, Japan). An elasticity tester (MPA580, Courage + Khazaka Electronic GmbH, Cologne, Germany) was used to test the elasticity and firmness of the facial skin, and the length, number, and depth of the facial skin wrinkles were tested using a skin pigment analyzer (Antera-3D, Miravex Limited, Dublin, Ireland). The skin melanin index was measured using a skin melanin and heme tester (Mexameter MX18, Courage + Khazaka Electronic GmbH, Cologne, Germany), and a skin color test probe (Colorimeter CL400, Courage + Khazaka Electronic GmbH, Cologne, Germany) was used to measure the skin color L value and ITA value. All tests were conducted at 21 ± 1 °C and 50 ± 10% R humidity, and the moisture content of the stratum corneum and the texture characteristics of each part of the face were verified. All tests were performed after the subjects cleansed their faces, and the corresponding data were recorded before the subjects used the GSO-ILE mask, after 30 min of using the GSO-ILE mask, and on the 7th, 14th, and 28th days of using the GSO-ILE mask.

### 3.13. Statistical Analysis

All data values are presented as the average and standard deviation. The significance of differences was assessed using one-way analysis of variance (ANOVA). The level of statistical significance was set at thresholds of * *p* < 0.05, ** *p* < 0.01, and *** *p* < 0.001. Herein, # indicates a *p*-value < 0.05, ### indicates 0.01 < *p* < 0.05, and #### indicates *p* < 0.001 when compared with the NC group.

## 4. Conclusions

The results of this study showed that the combination of Menthol-CoQ10 ionic liquid with nanotechnology provided grapeseed oil enhanced transdermal delivery efficiency and stronger efficacy. GSO-ILE presented a strong structure and content stability, better skin absorption, and deeper delivery during percutaneous penetration. At low concentrations, it scavenged DPPH free radicals and inhibited tyrosinase conversion. At the same time, the low concentration of GSO-ILE reduced the amount of melanin in melanoma cells and the production of MMP-1 by fibroblasts and increased the content of human type I collagen. In clinical experiments, GSO-ILE can significantly increase the water content of the stratum corneum; improve skin elasticity and firmness; reduce the degree of transepidermal water loss; and decrease the number and length of eye wrinkles, under-eye wrinkles, nasolabial folds, and marionette lines on the face. In summary, it can be seen that ionic liquids, as enabling agents, enhance the penetration and efficacy of oil, and their mechanism of action is that menthol in ionic liquids, as an enhancer, can change the lipid barrier of cells in the stratum corneum [36], increase the distribution of drugs from the water-soluble matrix to the stratum corneum, and interact with the skin epidermis and cause ultrastructural changes. At the same time, Coenzyme Q10, as an active ingredient with efficacy, can slowly improve the skin cell microenvironment, enhance the skin’s hydration ability [37], improve the skin barrier function, and then enhance the skin’s efficacy. In conclusion, the combination of ionic liquid and nanoparticles could be a promising method for oil delivery in cosmetic skincare area.

## Figures and Tables

**Table 1:** Seeding density of different cell types.

Cell Type	Human Melanoma Cells	Mouse Melanoma Cells	Human Keratinocytes	Human Fibroblasts
Seeding density (pcs/well)	1 × 10^4^	1 × 10^4^	1 × 10^4^	8 × 10^3^

## Data Availability

Data will be made available on request.

## Supplementary Materials

The following supporting information can be downloaded at: https://www.mdpi.com/article/10.3390/ph17101273/s1, Table S1: Summary of GSO-ILE mask human skin patch test results; Figure S1: Appearance of ionic liquid in grape seed oil; Figure S2: Menthol-Coenzyme Q10 ionic liquid NMR hydrogen spectroscopy results; Figure S3: Particle size stability of grape seed oil ionic liquid emulsion; Figure S4: Potential stability of grape seed oil ionic liquid emulsion; Figure S5: Content stability of coenzyme Q10 content in grape seed oil ionic liquid emulsion.
