# Supplementary material for: Ionic Liquid-Based Grapeseed Oil Emulsion for Enhanced Anti-Wrinkle Treatment
Source: Pharmaceuticals (Basel). 2024 Sep 26;17(10):1273. doi: 10.3390/ph17101273 (PMC11510344; doi:10.3390/ph17101273)
Supplement: Supplementary file 1 [file pharmaceuticals-17-01273-s001.zip › pharmaceuticals-3189790-supplementary.pdf]

Table S1 Summary of GSO-ILE mask human skin patch test results

Number 32	time	Negative control (blank matrix + membrane cloth)			GSO-ILE+Membrane cloth		
Grade	0.5	0（32）	0（32）	0（32）	0（32）	0（32）	0（32）
	24h	0（32）	0（32）	0（32）	0（32）	0（32）	0（32）
	48h	0（32）	0（32）	0（32）	0（32）	0（32）	0（32）

Note:0: Negative reaction, 1: Suspicious reaction, 2: Weak positive reaction, 3: Strong positive reaction, 4: Extremely strong positive reaction.

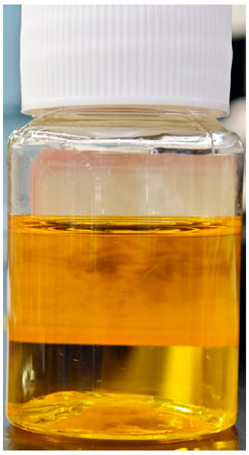

Figure S1 Appearance of ionic liquid in grape seed oil

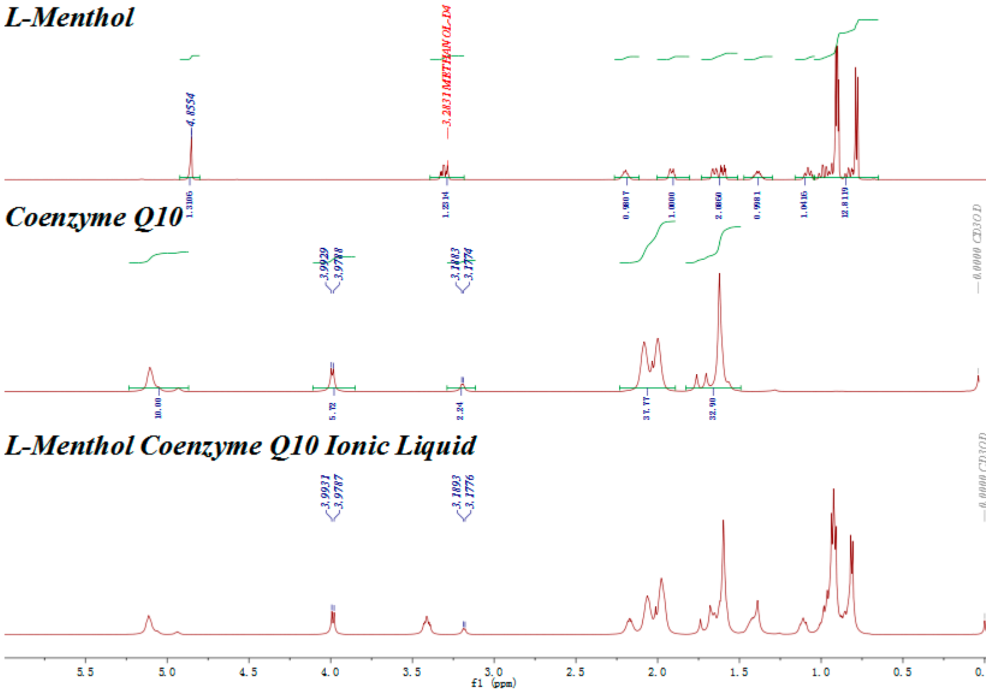

Figure S2 Menthol-Coenzyme Q10 ionic liquid NMR hydrogen spectroscopy results

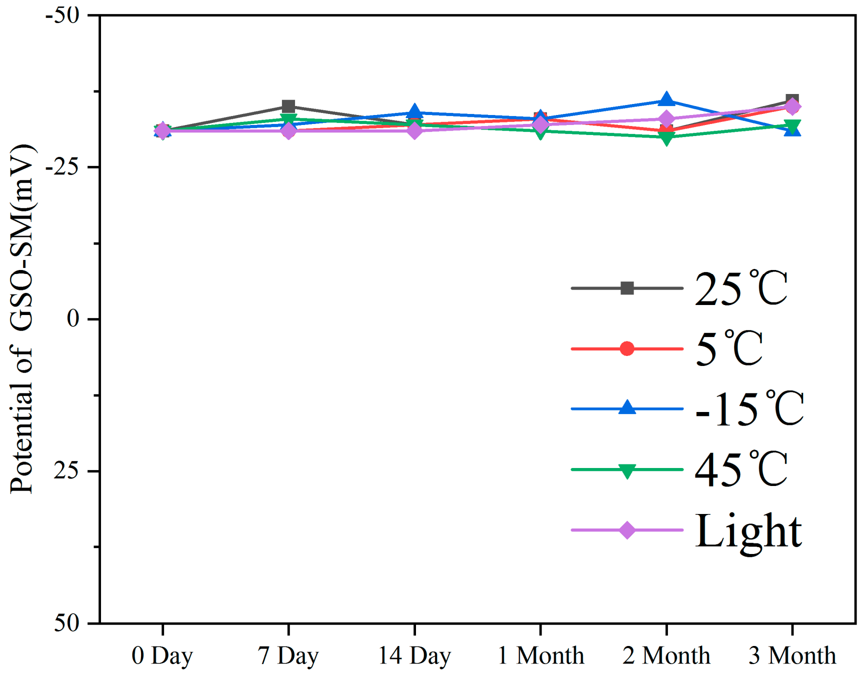

Figure S3 Particle size stability of grape seed oil ionic liquid emulsion

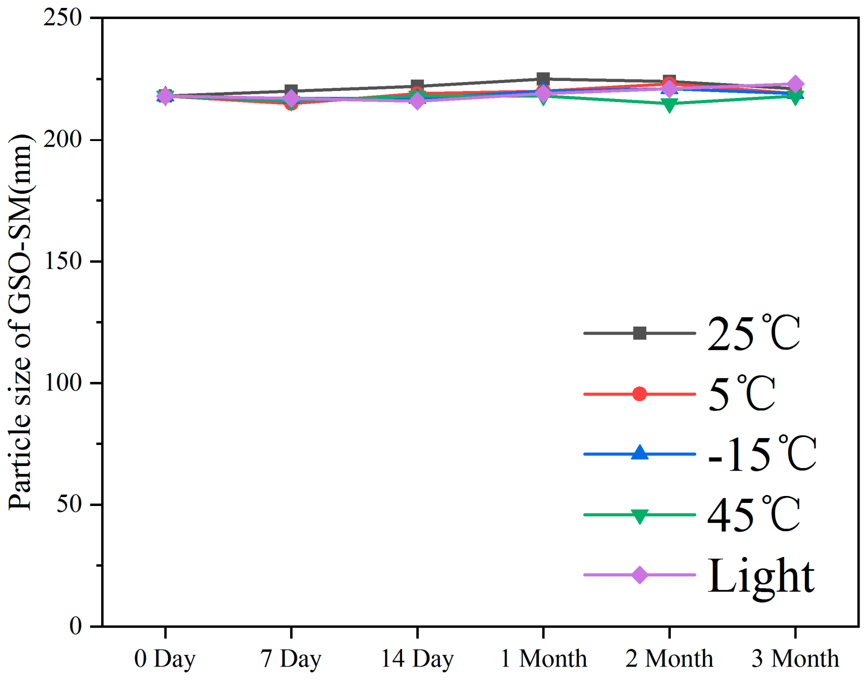

Figure S4 Potential stability of grape seed oil ionic liquid emulsion

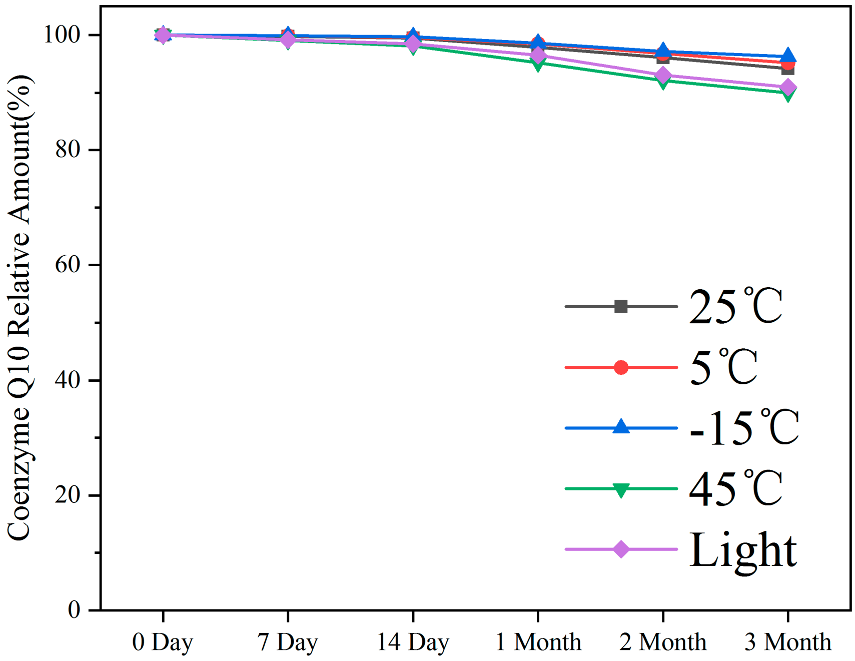

Figure S5 Content stability of coenzyme Q10 content in grape seed oil ionic liquid emulsion
